# Assessment of PlanIQ Feasibility DVH for head and neck treatment planning

**DOI:** 10.1002/acm2.12165

**Published:** 2017-08-30

**Authors:** David V. Fried, Bhishamjit S. Chera, Shiva K. Das

**Affiliations:** ^1^ Department of Radiation Oncology University of North Carolina at Chapel Hill Chapel Hill NC USA

**Keywords:** dosimetry, head and neck, optimization, PlanIQ

## Abstract

**Introduction:**

Designing a radiation plan that optimally delivers both target coverage and normal tissue sparing is challenging. There are limited tools to determine what is dosimetrically achievable and frequently the experience of the planner/physician is relied upon to make these determinations. PlanIQ software provides a tool that uses target and organ at risk (OAR) geometry to indicate the difficulty of achieving different points for organ dose–volume histograms (DVH). We hypothesized that PlanIQ Feasibility DVH may aid planners in reducing dose to OARs.

**Methods and materials:**

Clinically delivered head and neck treatments (clinical plan) were re‐planned (re‐plan) putting high emphasis on maximally sparing the contralateral parotid gland, contralateral submandibular gland, and larynx while maintaining routine clinical dosimetric objectives. The planner was blinded to the results of the clinically delivered plan as well as the Feasibility DVHs from PlanIQ. The re‐plan treatments were designed using 3‐arc VMAT in Raystation (RaySearch Laboratories, Sweden). The planner was then given the results from the PlanIQ Feasibility DVH analysis and developed an additional plan incorporating this information using 4‐arc VMAT (IQ plan). The DVHs across the three treatment plans were compared with what was deemed “impossible” by PlanIQ's Feasibility DVH (Impossible DVH). The impossible DVH (red) is defined as the DVH generated using the minimal dose that any voxel outside the targets must receive given 100% target coverage.

**Results:**

The re‐plans performed blinded to PlanIQ Feasibilty DVH achieved superior sparing of aforementioned OARs compared to the clinically delivered plans and resulted in discrepancies from the impossible DVHs by an average of 200–700 cGy. Using the PlanIQ Feasibility DVH led to *additional* OAR sparing compared to both the re‐plans and clinical plans and reduced the discrepancies from the impossible DVHs to an average of approximately 100 cGy. The dose reduction from clinical to re‐plan and re‐plan to IQ plan were significantly different even when taking into account multiple hypothesis testing for both the contralateral parotid and the larynx (*P* < 0.004 for all comparisons). No significant differences were observed between the three plans for the contralateral parotid when considering multiple hypothesis testing.

**Conclusions:**

Clinical treatment plans and blinded re‐plans were found to suboptimally spare OARs. PlanIQ could aid planners in generating treatment plans that push the limits of OAR sparing while maintaining routine clinical target coverage goals.

## 1 INTRODUCTION

Proper optimization of intensity modulated radiation therapy (IMRT) is central to providing treatments that provide conformal target coverage while sparing normal organs at risk (OARs). Technological advances in radiation treatment delivery have led to increases in plan complexity and increased variability in plan quality.^1^ Clinically, goal sheets are used routinely to guide IMRT planning in order to ensure that dosimetric constraints of OARs are met whenever possible. However, goal sheets do not explicitly provide information regarding the optimally achievable plan quality for a specific patient, but are rather a “one‐size‐fits‐all” generic recommendation for critical structure dose limits. To understand how these dose limits are applied in clinical practice, it is instructive to examine the IMRT clinical inverse planning process, which superficially seems quite simple. A planner dictates target doses and OAR constraints as inputs. An optimizer then finds the minimum of a cost function incorporating the desired doses and constraints. In actual practice, inverse planning is considerably more complicated; often requiring “dummy structures” such as avoidances, shells, and optimization structures along with multiple rounds of optimization to generate quality plans. Therefore, planning has become increasingly human/user dependent, which leads to increasing variability from plans between planners and even within departments/dosimetry teams. The final plan can also have considerable variability because of the planner's interpretation of the recommended dose limits—some planners may choose to stop sparing structures when the dose limit is just met, while others may choose to pursue further sparing after the dose limit is met. Automatic and knowledge‐based planning aids have been developed with the aim of increasing plan quality while reducing variability.

The variability in plans is particularly true for complex treatment sites such as the head and neck. This region of the body has an abundance of important and radiation sensitive OARs and frequently requires treatments of irregularly shaped planning target volumes (PTVs) with potentially multiple dose prescriptions. One major area of concern for head and neck treatments is the dosimetric sparing of a patient's salivary glands, pharyngeal constrictors, and larynx. High doses of radiation to these organs can cause dry mouth (xerostomia) and difficulty swallowing (dysphagia).^2^, ^3^, ^4^, ^5^, ^6^ Sparing a patient's salivary glands and larynx has been shown to reduce symptoms and increase patient quality of life.^2^, ^7^, ^8^, ^9^ The QUANTEC review of dose–volume effects on salivary function by Deasy et al. concluded that for IMRT plans the mean dose to each parotid gland should be kept *as low as possible*.^3^ It also states that, “a lower mean dose to the parotid gland usually results in better function, even for relatively low mean doses (<1000 cGy).”^3^ The same review examining larynx and pharynx dose–volume effects had a similar conclusion stating that planners should *minimize* the volume of pharyngeal constrictors and larynx receiving 6000 cGy, and when, possible 5000 cGy.^6^ Both publications emphasize the concept of minimizing the dose to these structures beyond the published/accepted benchmarks (i.e., as low as achievable). However, in practice, it is difficult to determine if a particular plan has in fact minimized the dose to these structures using a dose–volume histogram (DVH). The minimal dose to an OAR is predominantly dictated by the geometric relationship between the OAR and PTV(s). PlanIQ^TM^ software (Sun Nuclear, Melbourne, Florida, USA) offers a tool called *Feasibility DVH* ^*TM*^ which quantitatively determines regions of a DVH that are impossible (red), difficult (orange), challenging (yellow), and probable (green) on a per OAR basis, based on an ideal dose falloff from the prescription dose at the target boundary. The impossible DVH (red) is defined as the DVH generated using the minimal dose that any voxel outside the targets must receive given 100% target coverage. We performed a study comparing salivary gland and larynx sparing with and without the use of PlanIQ's Feasibility DVH. We studied whether PlanIQ's Feasibility DVH could provide accurate estimates of OAR sparing and whether its use during treatment planning could facilitate increased sparing of patients' salivary glands and the larynx while maintaining target coverage and overall plan quality.

## 2 MATERIALS AND METHODS

We identified 10 patients treated on one of two prospective protocols at our institution. All patients had primary lesions of the oropharynx and were node positive. Patients were originally treated using Tomotherapy (Accuray, Palo Alto, USA) (field width = 2.5 cm and pitch = 0.287–0.310) and retrospectively re‐planned using Raystation (RaySearch Medical Laboratories AB, Stockholm, Sweden) on an Elekta Versa HD (Stockholm, Sweden) using volumetric arc therapy (VMAT) with three full 6 MV arcs (re‐plan) and four full 6 MV arcs (IQ plan). The re‐plan treatments were performed blinded to the results of the clinical plan and PlanIQ Feasibility DVH. For the IQ plan, the results from the PlanIQ Feasibility DVH analysis were available and used during treatment planning.

During planning, the MLC leaf motion was limited to 0.48 cm/degree. The PTVs and OARs used during clinical planning were the same as used during the re‐plan and IQ plan. All OARs were expanded by 3 mm and PTVs pulled in skin 3 mm. A combination of equivalent uniform dose (EUD)‐ and DVH‐based planning methods were used. The high risk PTV (PTV HR) was prescribed to 6000 cGy and the standard risk PTV (PTV SR) was prescribed 5400 cGy in 200 cGy fractions. These two plans were delivered using simultaneous integrated boost (SIB). For the re‐plan, a multi‐criteria optimization (MCO) was generated for each patient followed by conversion to a deliverable plan and additional manual optimization. EUD optimization was used for sparing of OARs. Optimization structures were used for each OAR by subtracting the PTV SR with a 3 mm margin from the OAR. A maximum EUD (equation below) equal to 100–200 cGy less than the current average dose was used as a constraint with the “*a*” parameter equal to one prior to each round of optimizations.

(1) $$EUD={\left(\sum_{i}v_{i}\times {D_{i}}^{a}\right)}^{\frac{1}{a}}$$

where *v_i_* is the partial volume with absorbed dose *D*_*i*_.

This process was repeated until additional sparing was not achieved or sparing resulted in other plan issues such as hot spots, failing of clinical goals, etc. that could not be recovered. The goal of the re‐plan was to maximally spare the contralateral parotid, contralateral submandibular gland, and larynx while still meeting our routinely used clinical goal sheet (Table 1). For the IQ plan, the Sun Nuclear PlanIQ Feasibility DVH information was made available during the planning process. The same (MCO) was performed; however, rather than iteratively reducing the optimization constraints for the OARs, the mean value derived from the impossible DVH was used as the criteria for the max EUD with the “a” parameter equal to one. The EUD with the “a” parameter equal to one is equivalent to the mean dose. The re‐plan was performed blinded to the results of the clinically delivered plan as well as the Feasibility DVH information from PlanIQ. The IQ plans were performed aware of the Feasibility DVH information. The IQ plans were not compared to the clinically delivered plan nor the re‐plan during the planning process. A summary of the generated plans is shown in Table 2.

**Table 1:** Clinical goals for head and neck planning

Structure	Dosimetric goal	Dosimetric parameter
PTV HR	≥6000 cGy	V95%
PTV HR	≥5550 cGy	V99%
PTV HR	0.75	Conformity index^a^
PTV SR	≥5400 cGy	V95%
PTV SR	≥5022 cGy	V99%
PTV SR	0.75	Conformity index^a^
Brainstem + 3 mm	≥5400 cGy	Dose to 0.1 cc
Cochlea + 3 mm (applies to both cochleas)	≥4500 cGy	Mean dose
Cord + 3 mm	≥5000 cGy	Dose to 0.1 cc
Larynx + 3 mm	≥4100 cGy	Mean dose
Larynx + 3 mm	≥6000 cGy	D24%
Normal Tissue (skin minus PTV SR)	≥5940 cGy	D1%
Contralateral Parotid + 3 mm	≥2600 cGy	Mean dose
Contralateral Parotid + 3 mm	≥3000 cGy	D50%
Contralateral Submandibular + 3 mm	≥3500 cGy	Mean dose

a Defined as volume of the associated PTV divided by the volume of the prescription isodose line.

**Table 2:** Summary of generated plans

Plan type	Blinded	Treatment planning system	Machine	Optimization parameters
Clinical plan	N/A	Tomotherapy	Tomotherapy	DVH‐based
Re‐plan	YES	Raystation^a^	Versa HD	EUD/DVH‐based
IQ Plan	YES	Raystation^a^	Versa HD	EUD/DVH‐based

a Raystation used mutlicriteria optimization.

The Feasibility DVHs were generated by exporting the simulation CT and patient structures to PlanIQ. In PlanIQ, the PTVs were assigned their respective prescription doses and the Feasibility DVHs were calculated for 6 MV beams and a dose grid size of 2 mm using the maximal falloff method. The Feasibility DVHs are calculated using energy‐specific high gradient dose spread (HGDS) kernel assuming the entire PTV is receiving prescription. All neighboring voxels within a distance determined by the HGDS are searched, and if they are target surface voxels, their distance away (or radiological distance, if heterogeneity corrections are employed) and dose level produce a dose spread value at all nontarget voxels. A low dose spread (LDS) process adds a minimal low dose that must occur due to scatter and transmission assuming beams from many angles, 360° around the targets. Again, on a voxel‐by‐voxel basis, it estimates a minimal dose that must occur at any given voxel outside the targets, assuming 100% coverage and given the target sizes, shapes, and dose levels. Two low dose spread kernels are generated dynamically (based on nominal energy and target volume), one for near and far distances, and are used to convolve the original 3D target doses into two low dose 3D grids. There is energy‐dependent postprocessing of each LDS grid to further morph the lower dose values and to apply corrections in regions of low patient density. Then, for each LDS grid and for each nontarget voxel, the LDS value is compared to the current dose using the HGDS at all voxels, and if it is higher, then it replaces the current value in the benchmark grid. PlanIQ does not require any knowledge about the delivery technique or commissioning data.

The mean dose according to the impossible region of the Feasibility DVH was compared to the re‐planned and clinical mean dose for each of the three OARs mentioned previously. This comparison was performed postplanning for the re‐plan as it was blinded to the Feasibility DVH. The distribution of mean doses was not normally distributed based on the Shapiro–Wilk test of normality. Therefore, a paired Wilcoxon sign rank test was used to determine statistical significance between plans. A Bonferroni correction was used in order to account for multiple hypothesis testing (*P* < 0.008 was needed for significance).

All plans were determined if they would be “clinically deliverable” by the attending physician for these patients. The process of determining clinical deliverability was performed in the same manner as initial approval in that the physician reviewed the plan in all slices examining both DVH constraints as well as overall plan quality (i.e., no obvious hotspots outside the PTVs, reduced dose to the posterior neck and oral avoidances, etc.). To ensure that the generated plans were deliverable, our clinical IMRT QA procedure was performed on all IQ plans. A 3D diode array (ArcCHECK, Sun Nuclear, Melbourne, FL) was used to measure the machine delivered dose and SNC patient software (version 6.1.0; Sun Nuclear, Melbourne, FL, USA) was used to calculate the gamma pass rate using a global criteria of 3%/3 mm. At our institution, the standard criteria pass rate that we deem a plan acceptable for treatment is greater than 90%.

## 3 RESULTS

**Figure 1:**
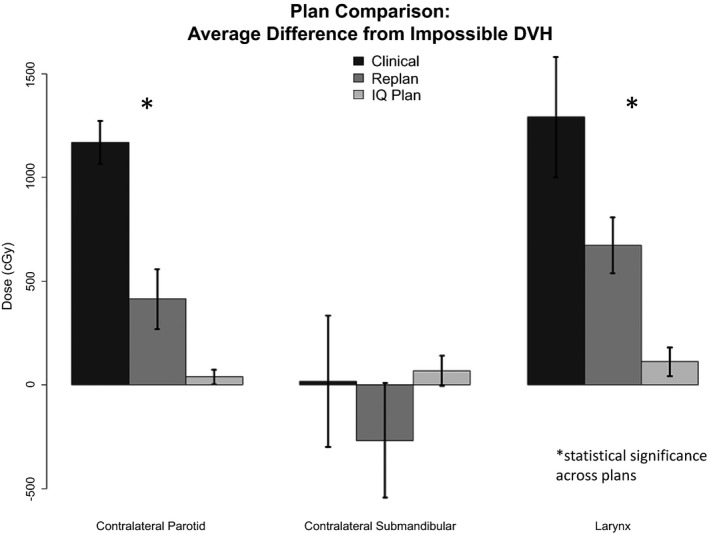
Comparing difference between clinical, re‐plan, and IQ plan versus Impossible DVH.

All plans passed our institutional IMRT QA standard (range: 92.8%–98.6%). The comparison between the mean dose from the clinical, re‐plan, and IQ plans to the impossible boundary from the Feasibility DVH (Impossible DVH) are shown in Fig. 1. The re‐plans were able to provide increased sparing of OARs compared to the delivered plans and subsequently agreed better with the mean dose from Impossible DVH. The contralateral parotid and larynx were spared for all patients in both the clinically delivered plan and the re‐plan. On average, the re‐plan reduced the dose compared to the clinical plan by approximately 750 cGy and 600 cGy for the contralateral parotid and larynx, respectively. For patients whose contralateral submandibular gland was spared in the clinical plan (7/10 patients), the re‐plans reduced the mean dose by approximately 300 cGy.

**Figure 2:**
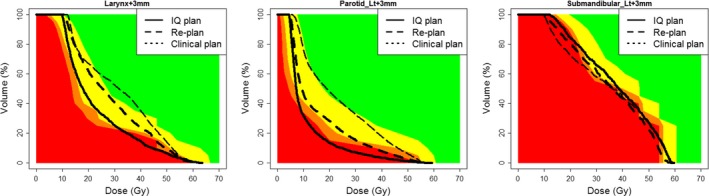
Patient 10 Feasibility DVH for larynx, contralateral parotid, and contralateral submandibular comparing the clinical, re‐plan, and IQ plan DVHs.

The IQ plans were found to reduce the mean dose to the contralateral parotid and larynx by approximately 1130 cGy (range: 890–1460) and 1180 cGy (range: 550–1890), respectively compared to the clinically delivered plans and 370 cGy (range: −20 to 690) and 560 Gy (range: 300–1090), respectively compared to the re‐plans. These values correspond to percent reductions of 50% and 34%, respectively compared to the clinically delivered plans and 24% and 19%, respectively compared to the re‐plans. The dose reduction from clinical to re‐plan and re‐plan to IQ plan were significantly different even when taking into account multiple hypothesis testing for both the contralateral parotid and the larynx (*P* < 0.004 for all comparisons). No significant differences were observed between the three plans for the contralateral parotid when considering multiple hypothesis testing. The sparing of the contralateral submandibular gland was relatively consistent across all plans. The average dose delivered to the larynx, contralateral parotid, and contralateral submandibular glands were on average within approximately 100 cGy of the predicted impossible DVH from PlanIQ. The DVHs for patient 10 compared to the Feasibility DVH are shown in Fig. 2 for each of the analyzed OARs.

## 4 DISCUSSION

The 10 retrospective re‐plans were performed in order to determine how close one could come to what PlanIQ deemed impossible using the Feasibility DVH. The average difference between re‐planned mean doses and the mean calculated from the Impossible DVH were 672 cGy, 416 cGy, and ‐248 cGy for the larynx, contralateral parotid, and contralateral submandibular gland, respectively. The IQ plans were then performed to determine if having the knowledge of Feasibility DVH during planning allowed for higher agreement and subsequently enhanced OAR sparing. We found that incorporating what the Feasibility DVH deemed the threshold of impossible into planning yielded dosimetry with very high agreement between planned and predicted sparing (<300 cGy for all patients and all OARs examined; approximately 100 cGy on average). Both the re‐plans and IQ plans found that substantial sparing was possible compared to what was clinically delivered. However, the fact that re‐plans were found to increase sparing is not surprising as they were performed without additional clinical load, time constraints, etc. that are experienced clinically by dosimetrists. What is surprising is the magnitude of the improvement in sparing between the clinical plans and the IQ plans. Reducing the dose to the contralateral parotid and larynx by ~1200 cGy could potentially lead to a meaningful improvement in patient toxicity/symptom burden.

This study demonstrates that planners could use the Feasibility DVH to guide dosimetric constraints during planning to ensure plans have been adequately optimized. Overall, the average difference between re‐planned and impossible DVH was found to be within 200–700 cGy for the salivary glands and larynx when blinded to PlanIQ's Feasibility DVH and approximately 100 cGy when this information is available during planning. The achieved doses were sometimes less than the Impossible DVH; however, this was found to be due to under dosing of the PTV (predominantly the PTV SR). As our routine prescription is to 95% coverage of PTVs, under dosing is permitted in OAR overlap regions while planning, but not accounted for in PlanIQ. This is why the re‐planned contralateral submandibular mean doses fall below the predicted impossible mean.

PlanIQ's Feasibility DVH could also be used as a plan quality tool where clinicians could make sure that plan DVHs fall within a certain range from the impossible threshold. The impossible DVH could also be a useful tool in determining what constraints cannot be met without compromising PTV coverage which would save planning time trying to optimize to constraints that are not achievable. Current practice relies on a single pass/fail Criterion when determining plan acceptability. Having a prediction of what is achievable would allow for constraints that are dependent on individual patient target/OAR geometry and would enable a higher degree of treatment personalization and optimization.

Other planning aids, such as RapidPlan^®^ and Autoplan^®^, are available to aid clinicians in a similar manner. However, knowledge‐based planning tools such as RapidPlan^®^ are dependent on the database of plans that are being used for reference. The plans included in model libraries may be consistent with what has been historically delivered, but may not be representative of what is dosimetrically achievable/optimal. A study by Tol et al. found that there were strong correlations between predicted and achieved mean doses when using RapidPlan.^10^ However, this does not address whether the data from these types of tools led to near‐optimal sparing of OARs. Autoplan^®^ is another aid designed to help planners to optimize treatments; however, realistic and achievable constraints are still necessary. PlanIQ's Feasibility DVH is based entirely on prescribed dose and contour geometry and therefore has no dependence on variation within a model library of previously generated plans. Our study demonstrated reasonable agreement between the PlanIQ impossible DVH and the IQ plans with average deviation being in the range of 100 cGy for the contralateral parotid, contralateral submandibular gland, and larynx. The ability to generate realistic constraints is beneficial on its own and may even improve the functionality of other planning aids such as RapidPlan^®^ and Autoplan^®^.

While PlanIQs calculations are only dependent on target/OAR geometry, beam energy, and CT density, they do not take into account delivery method or under dosing of PTVs. PlanIQ bases its calculations purely on maximum falloff from the target(s) and therefore may underappreciate difficulty in actual treatment delivery when many competing OARs are involved or if an OAR is particularly large (e.g., bone marrow, bowel space, etc.). The low dose spread components were fit based on phantom cases and validated, in part, based on the methods by Ahmed et al. (in press) (i.e., optimize a plan to give 100% coverage and spare only one critical OAR at a time, then compare the achieved DVH to Impossible DVH). Our results found that PlanIQ performed well in head and neck cases at providing useful information that planners could use to increase plan quality even in the presence of multiple OARs. It may be unrealistic to think *all* OARs will have attainable DVHs in close proximity to their corresponding impossible DVH but the data do show that the impossible DVH does represent a good baseline for possible sparing. Furthermore, it was noted that additional dose was given to regions of the posterior skull base, mandible, and posterior neck in the IQ plans compared to the clinically delivered plans. The clinical plans were also found to have less heterogeneity within the PTVs compared to the IQ plans. However, no plans contained a PTV HR hot spot greater than 109% of the Rx. Nevertheless, all plans were considered clinically deliverable by the attending physician for these patients.

The fact that PlanIQ does not consider beam delivery can be seen as a positive in some ways when comparing to knowledge‐based tools. Knowledge‐based planning may lose accuracy if the implemented delivery is different from the treatments included in the model library, whereas calculation‐based methods such as PlanIQ's Feasibility DVH do not have this added component of variability.

An additional cofounding factor besides clinical load, time constraints, etc. that makes comparison between the clinical plan, re‐plan, and the IQ plan difficult is that the two were planned on different machines (Tomotherapy versus Versa) and different treatment planning systems (Tomotherapy versus Raystation). Tomotherapy was used to treat all patients in this cohort originally and our institution has subsequently has shifted to predominantly using Raystation and therefore this was of more clinical interest. In terms of the results, we view that our major finding is not the quantitative reduction in sparing achieved between the clinical to IQ plans, but the proximity by which the IQ plan adheres to the predictions provided by the feasibility DVH (<2 Gy). The purpose of including the clinical plans was to illustrate that plans that pass our routine clinical goals and are deemed appropriate for patient delivery can often be improved. Anecdotally, in our clinical practice we have not observed drastic discrepancies plan quality or ability to achieve Feasibility DVH criteria between Tomotherapy planning versus Raystation/Versa planning. The IQ plans were shown to be deliverable and provided additional sparing of the salivary structures and larynx even when compared to the re‐plans using the same treatment planning system and machine. These results realistically present two different planning paradigms that need clinical consideration. The clinical plans were delivered with more homogeneous target doses and better low dose conformity, whereas the IQ plans had superior OAR sparing but more heterogeneous target doses and inferior low dose conformity in certain areas. Our institution is a high volume, academic center that is an active participant in head and neck clinical trials. Despite these credentials, we were surprised to see how much higher our OAR doses were compared to those predicted by PlanIQ. Subsequently, the IQ plans were able to demonstrate that these predictions were quite accurate in terms of what could be delivered. These results have led us to begin incorporating PlanIQ into our routine clinical planning processes.

In the future, our institution hopes to continue to utilize PlanIQ and to standardize methods by which this information is incorporated into the planning process. While we utilized 3 and 4‐arc VMAT for this research, we have yet to determine if similar plans could be generated with few arcs in order to optimize clinical throughput. We also intend to investigate whether PlanIQ can not only improve plan quality but also if it may result in a reduction of planning time by providing reasonable estimates of expected DVHs upfront in the planning process. Evaluation of PlanIQ needs to be performed for sites other than head and neck.

Tools capable of providing predictions of what is dosimetrically achievable (and ideally optimal) are greatly needed in radiation treatment planning in order to reduce plan variability and ensure quality. This work demonstrates for the first time that PlanIQ's Feasibility DVH agrees well with head and neck treatment plans that attempted to maximally spare salivary glands and the larynx. The addition of the Feasibility DVH information during planning led to an increased sparing of OARs compared to both clinical plans and plans blinded to this information. This suggests the Feasibility DVH could be a useful tool during planning and as a plan quality assurance tool. In the future, quantitative predictions such as the Feasibility DVH may be used in tandem with knowledge‐based or auto‐planning to provide the best of both worlds in terms of a tool that can address Feasibility, optimality, and deliverability. Additional studies are needed examining the incorporation of Feasibility DVHs during treatment planning and whether it could also lead to increases in clinical efficiency.

## CONFLICT OF INTEREST

Departmental research agreement with Sun Nuclear.
